# Supplementary material for: A Critical View of the Application of the APEX Software (Aqueous Photochemistry of Environmentally-Occurring Xenobiotics) to Predict Photoreaction Kinetics in Surface Freshwaters
Source: Molecules. 2019 Dec 18;25(1):9. doi: 10.3390/molecules25010009 (PMC7017383; doi:10.3390/molecules25010009)
Supplement: Supplementary file 1 [file molecules-25-00009-s001.zip › 3.2.4_gcc-4.4.0.zip!/3.2.4_gcc-4.4.0/3.2.4_gcc-4.4.0/doc/octave/html/interpreter/Logical-Values.html]

Logical Values - Untitled


Next: Promotion and Demotion of Data Types,
Previous: Bit Manipulations,
Up: Numeric Data Types


---

### 4.6 Logical Values

Octave has built-in support for logical values, i.e., variables that
are either `true` or `false`. When comparing two variables,
the result will be a logical value whose value depends on whether or
not the comparison is true.

The basic logical operations are `&`, `|`, and `!`,
which correspond to “Logical And”, “Logical Or”, and “Logical
Negation”. These operations all follow the usual rules of logic.

It is also possible to use logical values as part of standard numerical
calculations. In this case `true` is converted to `1`, and
`false` to 0, both represented using double precision floating
point numbers. So, the result of `true*22 - false/6` is `22`.

Logical values can also be used to index matrices and cell arrays.
When indexing with a logical array the result will be a vector containing
the values corresponding to `true` parts of the logical array.
The following example illustrates this.

```
     data = [ 1, 2; 3, 4 ];
     idx = (data <= 2);
     data(idx)
          => ans = [ 1; 2 ]
```

Instead of creating the `idx` array it is possible to replace
`data(idx)` with `data( data <= 2 )` in the above code.

Logical values can also be constructed by
casting numeric objects to logical values, or by using the `true`
or `false` functions.

— Function File: **logical** (arg)  
> Convert arg to a logical value. For example,
>
> ```
>           logical ([-1, 0, 1])
> ```
>
> is equivalent to
>
> ```
>           [-1, 0, 1] != 0
> ```

— Built-in Function: **true** (x)  
— Built-in Function: **true** (n, m)  
— Built-in Function: **true** (n, m, k, ...)  
> Return a matrix or N-dimensional array whose elements are all logical 1.
> The arguments are handled the same as the arguments for `eye`.

— Built-in Function: **false** (x)  
— Built-in Function: **false** (n, m)  
— Built-in Function: **false** (n, m, k, ...)  
> Return a matrix or N-dimensional array whose elements are all logical 0.
> The arguments are handled the same as the arguments for `eye`.
